# Self-healable soft shield for γ-ray radiation based on polyacrylamide hydrogel composites

**DOI:** 10.1038/s41598-020-78663-x

**Published:** 2020-12-10

**Authors:** Jinwoo Park, Minseok Kim, Sooseok Choi, Jeong-Yun Sun

**Affiliations:** 1 Department of Material Science and Engineering, Seoul National University, Seoul, 08826 South Korea; 2 Research Institute of Advanced Materials (RIAM), Seoul National University, Seoul, 08826 South Korea; 3 Department of Nuclear and Energy Engineering, Jeju National University, Jeju, 63243 Republic of Korea

**Keywords:** Materials science, Nanoscience and technology

## Abstract

With the growing risk of radiation exposure, there are growing interests in radiation shielding. Because most radiation shields are made from heavy metals, a need to develop a soft shield is raised to protect human body. However, because the shield can easily undergo a mechanical damage by an impact, it would be better to have self-repairing system in the shield. Here, we have fabricated an intrinsic self-healable soft shield for gamma ray by making acrylamide based hydrogel composite. The composite contains lead dioxide nanoparticles for gamma ray shielding and Laponite clays for self-repairing. Although the hydrogel contained a large amount of lead dioxide nanoparticles (3.23 M), the fabricated composites stretched beyond 1400% while showing a high attenuation coefficient of 0.1343 cm^−1^ against gamma ray from a cobalt-60 source. Then a systematic study was performed to analyze self-healing properties and the 96.55% of maximum self-healing efficiency was obtained. We also analyzed a storage modulus of hydrogel and molecular weight of polyacrylamide to study an effect of gamma ray on the self-healing. The self-healing efficiency was decreased by a gamma ray because the radiation induces scissioning or covalent crosslinking in the chains.

## Introduction

Radiation poisoning refers to an acute illness caused by exposure to a high dose of ionizing radiation^1^. Precise amounts of ionizing radiation are used in a wide variety of fields, such as in medicine, but out-of-range radiation poisoning can cause problems including sterility, leukemia and death^2,3^. Various types of ionizing radiation can be produced, including alpha, beta, gamma radiation, neutron particles, and X-rays. Among these, gamma rays are of most concern since gamma rays are intense enough to penetrate inside the human body. Therefore, there is an increasing need to protect the human body from exposure to gamma radiation. Recently, research has been conducted on shields for gamma rays in various applications, such as space engineering, nuclear power generation, and the military industry^4^.

Typically, a radiation shield is composed of metals with high atomic number and density, such as lead, tungsten, and tin, to block the transmission rate of radiation. However, such heavy metals are too heavy, rigid and bulky to wear to be able to efficiently protect the wearer. A soft shield on the other hand would be able to wear easily and deform during use. One way to develop a soft shield is through the synthesis of a composite mixing small particles into a stretchable matrix. For such particles, it is important to maintain their properties as a radiation shield while also forming a uniform composite with the matrix. At the same time, the matrix should provide excellent mechanical toughness so that it is not damaged when it contains a large amount of particles while also remaining safe to wear on the skin. To satisfy these requirements, rigid metal shields are becoming replaced with more versatile materials that incorporate metal nanoparticles into a polymer matrix in a hydrogel^5^.

Hydrogels are crosslinked polymer networks that contain a high water content^6^. Hydrogels are very stretchable, biocompatible and soft^7,8^. Furthermore, the free volume of water between the tough polymer matrix can encapsulate plenty of nanoparticles. Therefore, hydrogels are suitable to manufacture composites that are combined with nanoparticles and encapsulate nanoparticles. For example, hydrogel-metal nanoparticle composites with sewability and a high attenuation coefficient (0.28 cm^−1^) were achieved by adding lead dioxide nanoparticles into hydrogel^9^. In this way, the rigid metal shield can be replaced with a soft shield for use in various applications. However, unlike bulky metals that can be attached or repaired by welding, hydrogel metal nanoparticle composites are generally susceptible to rupture. Therefore, several researches have been conducted to increase their durability and stability.

One way to enhance durability of a hydrogel is designing it with self-healing properties. Self-healing is the ability of the material to autonomously heal or repair itself^10^. By creating hydrogel composites with this ability, damaged composites can be reused to extend their life, and multiple composites can easily be joined to make a larger piece. Therefore, self-healing hydrogel research has been actively conducted, and various self-healing mechanisms have been found. For instance, highly stretchable (~ 3600%) self-healing hydrogels with self-healing efficiency of 100% were formed with hydrophobic parts made of micelles^11^. Also, a disulfide based hydrogel with more than 50% self-healing efficiency was reported to have self-healing induced under acidic and basic conditions^12^. However, both of these self-healing mechanisms are not suitable for use with hydrogel-metal nanoparticle composites for radiation shielding since the formation of micelles is hindered by the large amount of metal-nanoparticles, and disulfide bonds are vulnerable to radiation energy, so cleavage occurs even with UV energy^13^. The other self-healing mechanism consists of a nanocomposite with a polymer–nanoclay network^14^. The hydrogen bonds between the clay and polymer that induces self-healing can be broken by gamma radiation. However, the bond can be restored if the bonding sites are still alive. Moreover, there is sufficient free volume in the clay-polymer hydrogel composites to contain a large quantity of nanoparticles. Therefore, it is possible to develop a soft shield that can sufficiently block a gamma ray radiation.

There have been efforts to incorporate self-healing into the radiation shielding composites. For example, studies on self-healing soft electromagnetic shielding materials for low frequencies of several hundred GHz have been developed^15–19^. However, self-healing soft materials that can shield the strong energy of gamma ray have been still required. Another self-healing soft shield based on PVA–Bi_2_O_3_ is for shielding gamma ray radiation^20^. Although the hydrogen bond based self-healing hydrogel composite has an elongation of 560%, HVL of 5.75 cm and self-healing efficiency of 75%, elongation and self-healing efficiency are still insufficient to increase durability of hydrogel composites and apply to wearable form.

Here, we have developed highly stretchable and self-healing hydrogel composites fabricated from acrylamide (AAm) polymer with lead dioxide (PbO_2_) nanoparticles and crosslinked with a nanoclay as shield materials against gamma ray radiation. The PbO_2_ nanoparticles have a very high attenuation coefficient, stability against oxidation, and a size of several tens of nanometers^9^. Therefore, PbO_2_ nanoparticles were used to absorb the gamma rays, and a Laponite nanoclay–polyacrylamide combination was used to provide the self-healing properties to the soft shield. Through this, we intend to overcome the shortcomings of the heavy metallic shield and propose an alternative soft shield which can be applied in a wearable form. Then, the effect of variations in the processing factors was studied to systematically maximize the self-healing properties of the composite with a fixed PbO_2_ concentration with highest attenuation coefficient^9^. In addition, the radiation shielding performance of the hydrogel composites was determined using a radiation source of cobalt-60 (Co-60) (1.25 meV). We also studied the effect of the amount of radiation that could be fatal to the human body (> 10 Gry) on self-healing efficiency. By investigating the change of hydrogel and acrylamide according to the radiation exposure, we identified causes that affect self-healing efficiency.

## Results and discussion

### Synthesis of self-healable γ-ray shielding hydrogel composite

**Figure 1:**
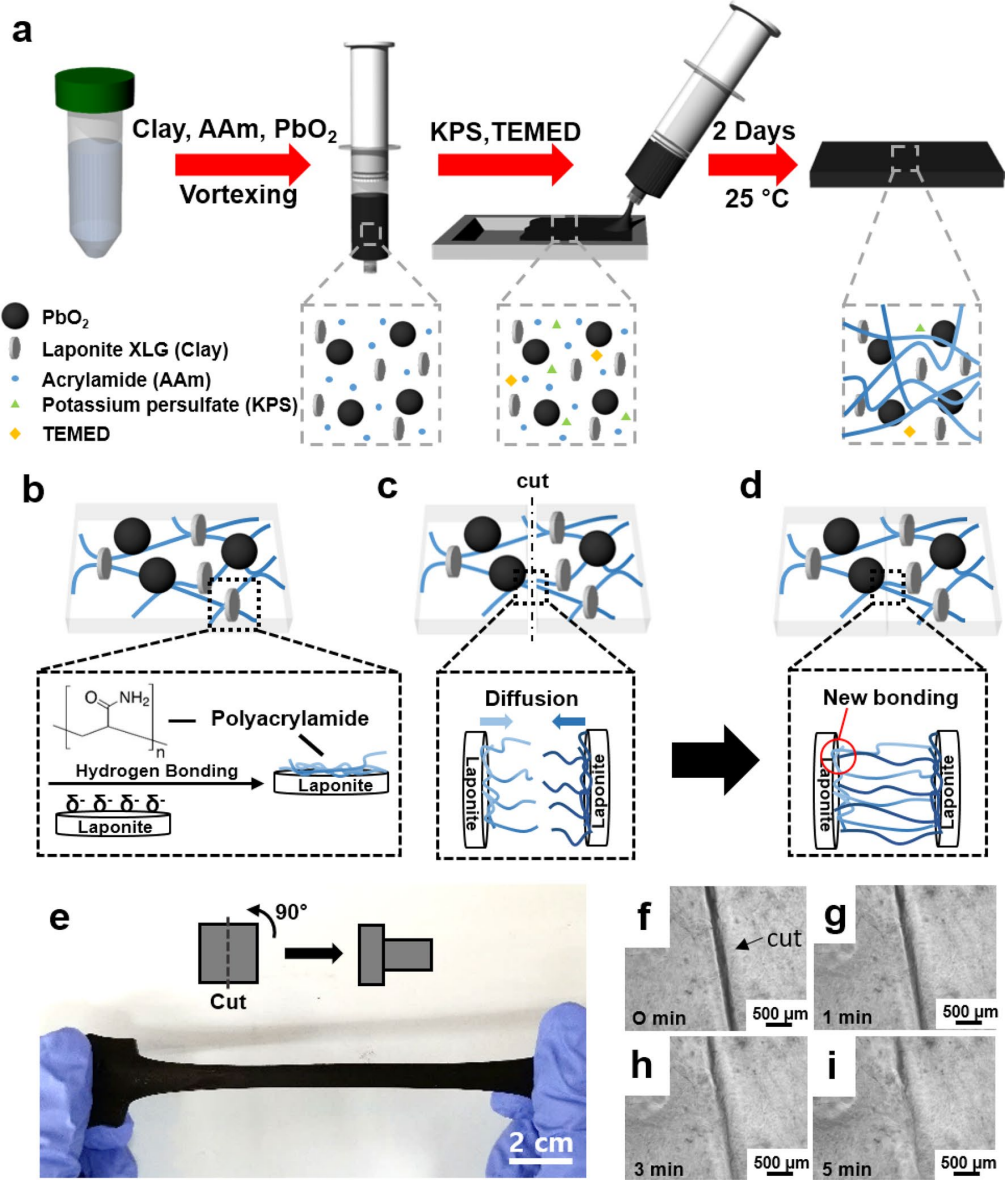
Self-healable γ-ray shield hydrogel. (**a**) Fabrication process of highly stretchable and self-healing hydrogel-metal oxide composite for γ-ray radiation. (**b**) Schematic illustration of internal structure of hydrogel composites. Polyacrylamides chains are adsorbed on the surface of Laponite due to hydrogen bonding. (**c**, **d**) A cut polyacrylamide chains can form a new hydrogen bonding with a Laponite by thermal diffusion. (**e**) Gel cut in half and healed after rotating one piece 90°. The gel was elongated after healing at 55 °C for 2 h. (**f**–**i**) Optical microscope images of fast self-healing behavior of cut surfaces up to 5 min at room temperature.

We synthesized stretchable and self-healable radiation shielding hydrogel composites by combining an acrylamide polymer with clay containing PbO_2_ nanoparticles. The fabrication process for the gel composites follows regular thermal radical polymerization, as shown in Fig. 1a. Despite the large amount of PbO_2_ nanoparticles, 3.23 M, the polymer chains were effectively combined with the clay, resulting in homogeneous polymerization. In addition, there were no leakage of metal nanoparticles from hydrogel composites. Specifically, the polymerization process is as follows. When a clod of Laponite clay was submerged in water, the clay became hydrated and swollen. So that the clay was divided into disk-like particles with a thickness around 1 nm^21^. On the surface of the clay, which is evenly distributed in the water, partially negative charges are widely distributed due to numerous silanol groups. Therefore, the interaction between the amide side groups on the polymer and the surface of the clay is ascribed to hydrogen bonds, resulting in polymer aggregation on the surface of the clay and polymerization. Finally, the formation of the hydrogel via physical crosslinking is completed (Fig. 1b). When the gel was cut and separated, even though some part of polymer chains were broken by the cutting, there are still remained grafted polymer chains on the surface of the clay. By attaching the two cut surfaces together, the grafted polymer chains diffuse into the other side of the cut gel and form additional hydrogen bonds. Because the state of physical crosslinking becomes similar to before cutting, self-healing is accomplished (Fig. 1c,d).

To demonstrate the healing ability of the gel, a size of 30.0 × 30.0 × 2 mm^3^ self-healable gel with 35.6 wt% clay/AAm ratio and 3.23 M lead dioxide was prepared. Then, the gel was cut and contacted to make a T-shape and treated for 2 h at 55 °C. The T-shaped gel was stretched after healing (Fig. 1e) demonstrating its healing abilities. Furthermore, hydrogels were fabricated without PbO_2_ nanoparticles to observe the real-time self-healing process of the hydrogel composites monitored by using an optical inverted microscope (Olympus Korea., CKX41-A32PH) (Fig. 1f–i). The optical microscopy images showed self-healing behavior on adjacent surfaces for 1–5 min at room temperature. Consequentially, we could confirm that the healing process was carried out very quickly and spontaneously without external stimulus, which is also referred to as intrinsic self-healing^22^.

### Self-healing performance of PAAm–Laponite–lead dioxide hydrogel composite

The self-healing mechanism is affected by diffusion of polymer chains. The curvilinear motion of the polymer chain is expressed in the following diffusion equation^23^:

1 $${D}_{c}=\frac{{k}_{B}T}{N\xi }$$

where ${D}_{c}$ is curvilinear diffusion, ${k}_{B}$ is the Boltzmann constant, *N* is Kuhn segments per polymer chain, $T$ is the absolute temperature, and $\xi$ is the friction coefficient due to topological interactions. The diffusion is influenced by the polymer chain length, distance between the clays and the temperature. Specifically, an increase in the chain length causes an increase in *N*, and the high clay concentration reduces the distance between the clays and the free volume in the hydrogel, resulting in high $\xi$ values in the hydrogel^24,25^. Hence, a longer healing time is required as ${D}_{c}$ decreases. Furthermore, as the increase in temperature leads to smaller $\xi$ and increasing $T$, healing is accelerated due to increase in ${D}_{c}$. For these reasons, the proper ratio and amounts of AAm and clay with a high temperature are necessary to maximize the self-healing properties of the hydrogel. Therefore, the self-healing property was verified by modulating the healing times, healing temperature, percent ratio of clay/AAm, and weight percent of water. To modulate weight percent of water, the amount of clay and acrylamide was increased proportionally by a certain percentage ratio (clay/AAm of 35.6%). In the particular case of adding more than a certain amount of clay, clays form a house of card structure in water and gelation occurs naturally^26^. This phenomenon hinders the even distribution of substances in composites. Therefore, the synthesis of composites was made up to the clay content before the gelation occurred.

**Figure 2:**
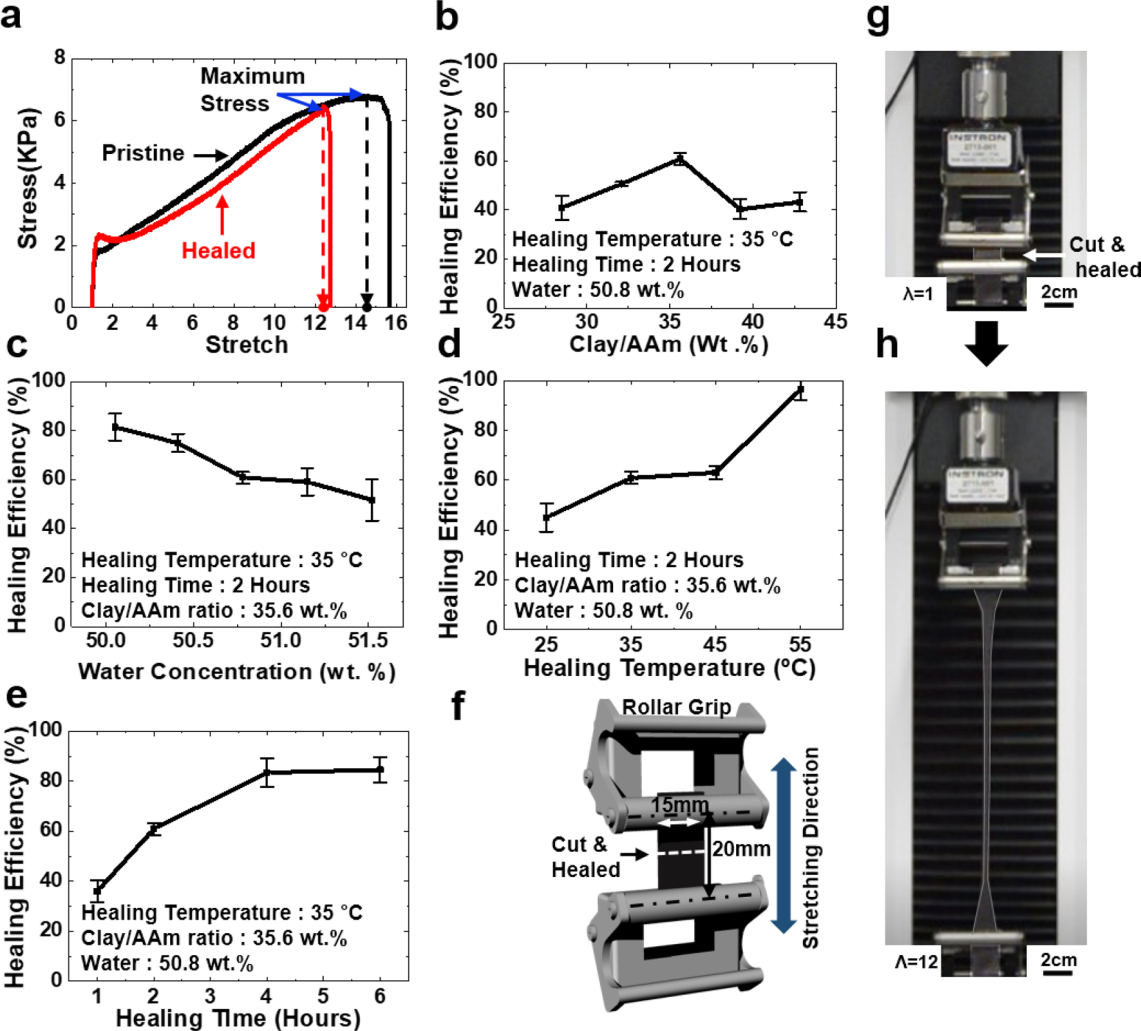
Self-healing performance of PAAm–Laponite–lead dioxide hydrogel. The molecular weight of lead dioxide is fixed as 3.23 M. (**a**) Tensile stress–strain curves of a pristine and a self-healing hydrogel containing 35.6 wt. % of Laponite versus PAAm. The sample was healed at 35 °C for 4 h. (**b**–**e**) The efficiency of self-healing in various conditions. Ratio between clay and PAAM, water weight percent, healing temperature and time were investigated. (**f**) Geometry of a grip and a sample for a tensile test. (**g**, **h**) Self-healing hydrogel is stretched up to 12 times of its initial length in a tensile machine. The gel has 35.6 wt% of clay/AAm ratio and 50.8 wt% of water content. The gel was healed at 35 °C for 4 h. Error bars show standard deviation; sample size n = 6.

Figure 2a shows stress–strain curves of the hydrogel composites. A damaged gel was almost fully recovered from a cut by a self-healing because the healed gel showed very similar stress–strain curves to pristine gels. To determine the self-healing efficiency, the criteria of the maximum strain was defined as the strain value corresponding to the maximum stress of the hydrogel composites shown in Fig. 2a, which is marked with dots on the x-axis. Then, the self-healing efficiency is defined according to the Healing Efficiency (%) = (maximum strain of healed gel/maximum strain of pristine gel) $\times$ 100. To examine the effects of the clay/AAm ratio on the self-healing, the hydrogel composites were prepared with different clay/AAm ratios that were adjusted by changing the amount of AAm at a fixed clay amount (3.61 wt% to distilled water) (Fig. 2b). In addition, we fabricated 35.6% clay/AAm ratio gel with various amounts of clay and AAm to the water weight percent (Fig. 2c). The samples were treated at 35 °C for 2 h. The self-healing efficiency initially increased with an increase in the clay/AAm ratio and reached a maximum of 60.89% at 35.6% followed by a decrease. Furthermore, the self-healing efficiency was shown to gradually decrease as the overall water weight percent was reduced. These suggest the contribution of clay and acrylamide on self-healing, which plays a major role in the hydrogen bond. In addition, the balance of clay-AAm also has a great effect on the self-healing efficiency by influencing the length of the polymer chain between clays. Thereafter, the impact of healing time and temperature on the self-healing was examined to investigate the contribution of hydrogen bonding to self-healing properties by changing the healing temperature (25 °C, 35 °C, 45 °C, 55 °C) and healing times (1 h, 2 h, 4 h, 6 h). As shown in Fig. 2d and e, the healing efficiency generally increased with an increase in temperature and healing time, especially the maximum healing efficiency of 96.55% was achieved at 55 °C for 2 h. Figure 2f shows the geometry of tensile test specimen used to measure self-healing efficiency. The gel is containing 3.23 M of lead dioxide with 35.6% clay/AAm ratio and 50.8 wt% of water. The specimen was adjusted to size of 15.0 $\times$ 20.0 $\times$ 2 mm^3^, healed at 35 °C for 4 h after introducing a cut in the middle. The healed hydrogel composite was then stretched up to 12 times of its original length before a rupture (Fig. 2g,h).

### Gamma ray exposure tests on radiation shielding hydrogel composite

**Figure 3:**
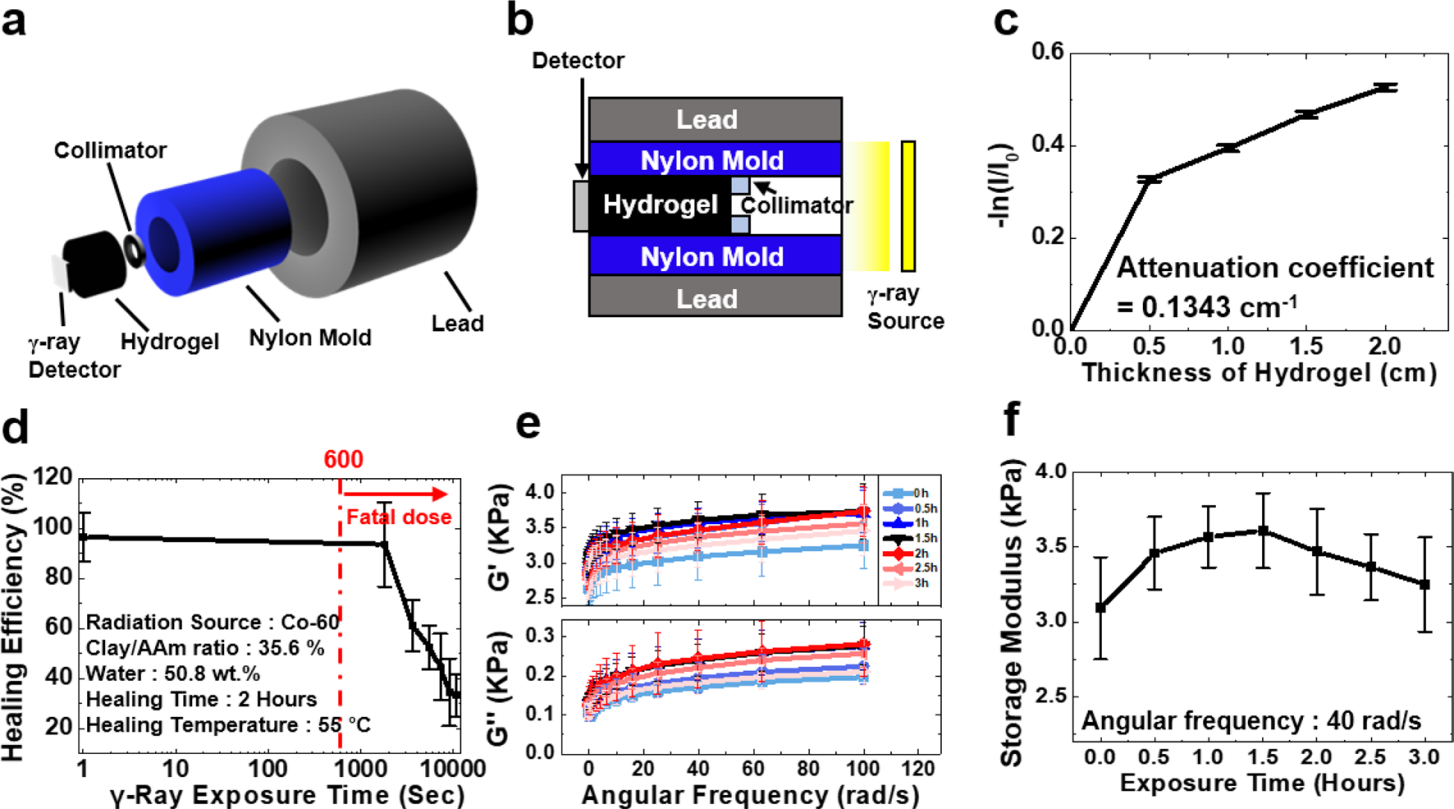
Γ-ray exposure tests on radiation shielding hydrogel composite. (**a**, **b**) Schematic illustration of experimental apparatus for a measurement of the γ-ray transmission. (**c**) Measurement of logarithmic-scale of transmittance according to thickness of the composites. Attenuation coefficient was calculated using the Beer Lambert Law. Error bars show standard deviation; sample size n = 3. (**d**) Self-healing property of the gel by exposure time. The red dotted line indicates the minimum fatal radiation dose to humans (10 Gy). (**e**) The effect of γ-ray exposure time on PAAm hydrogel expressed as storage and loss modulus graph. The gel does not contain any Laponites and lead dioxides. Error bars show standard deviation; sample size n = 6. (**f**) Storage modulus of PAAm hydrogel at an angular frequency of 40 rad/s.

Properties of radiation shielding of the gel composites were evaluated in Fig. 3. To measure the degree of shielding, an experimental apparatus made of nylon mold was produced. A cylinder shape thick lead is surrounding the nylon mold to block an unexpected γ-ray exposure. Then, gels with various thickness (5 mm, 10 mm, 15 mm, 20 mm) were located after a collimator to measure the radiation intensity through a detector by emitting γ-ray radiation (Fig. 3a,b). The transmittance (%) was measured as the ratio of the measured radiation intensity before and after loading a gel. An attenuation coefficient was calculated by fitting the transmittance curve with Beer Lambert Law^27^,

2 $$I/{I}_{0}={e}^{-\mu x}$$

where ${I}_{0}$ is the incident γ-ray intensity, $I$ is the defected which is measured with a shield of thickness $x$, and $\mu$ is the attenuation coefficient. As shown in Fig. 3c, the logarithmic-scale of transmittance decreased steadily with increasing thickness. Based on the graph, an attenuation coefficient of 0.1343 cm^−1^ was obtained. In order to evaluate performance of the shield, the tenth-value layer (TVL) and half-value layer (HVL) values, which mean the required thickness of the material at which the intensity of radiation entering is reduced by 90% and 50% respectively, are calculated by Eqs. (3) and (4)^28^.

3 $$TVL=ln10/\mu$$

4 $$HVL=ln2/\mu$$

The TVL value of 17.15 cm and HVL value of 5.16 cm were obtained. The values are comparable to that of ordinary concrete, which has a TVL value of 17.26 cm and HVL value of 5.19 cm^29^, and the HVL value of other reported results are in Table 1.

**Table 1:** HVL value of radiation shielding materials at 1.25 meV.

Shielding material	Polymer	Attenuation coefficient (cm^−1^)	HVL (cm)	Ref.
Water	–	0.06323	10.96	^29^
Ordinary concrete	–	0.13342	5.19	^29^
Bi_2_O_3_	Nature rubber	0.105	7	^30^
Bi_2_O_3_	Polyvinyl alcohol	0.127	5.475	^20^
CdO	High density polyethylene	0.104	6.67	^31^
Pb_3_O_4_	Phenyl epoxy	0.103	6.74	^32^
PbO_2_	Polyacrylamide	0.1343	5.16	This work

The effect of radiation on self-healing was investigated in Fig. 3d. The healing-efficiency decreased dramatically after 1800 s. Although this result showed that the self-healing mechanism is negatively affected by large dose of radiation exposure, the composite is enough to be used to protect the body under a mild dose^33^. A fatal dose of 10 Gy for human beings is converted to 600 s of γ-ray exposure in a distance of 30 cm with 1.25 meV Co-60 source. To determine a reason of the decrease of self-healing property due to the radiation, a hydrogel with clay/AAm ratio of 35.6%, water content of 50.8 wt% was fabricated. In order to maximize the effect of radiation, PbO_2_ nanoparticles were not added to the hydrogel. Then, the hydrogel was exposed to the range 0–300 Gy of radiation. By using complexometric titration experiments with ethylenediaminetetraacetic acid (EDTA), it is possible to determine whether the Laponite has collapsed due to radiation^34^. If the Laponite is decomposed, a color change is observed which may be induced by Mg^2+^ leakage. From this experiment, no changes in color of the titrant was observed resulted from the collapse of Laponite.

The effects of γ-ray radiation in polymer chains were explored by synthesizing hydrogels with *N*,*N*′-Methylenebisacrylamide (MBAAm) as a chemical crosslinker. Chemical hydrogels were exposed to radiation by time. Then storage and loss modulus data of hydrogels were obtained from the rheometer. Figure 3e shows the rheometer graph of the MBAAm crosslinking hydrogels, and Fig. 3f shows the changes in the storage modulus with exposure time at angular frequency of 40 rad/s. As shown in the graph, the storage modulus of the hydrogels initially increases and then decreases with the exposure time. This tendency indicates that chain cross-linking in the hydrogels was occurred and then broken. To analysis and compare with this tendency, dried polyacrylamides were obtained with a freeze-dryer. The value of average molecular weight was analyzed with Gel Permeation Chromatography (GPC) to verify the deformation of the polymer chains. The result and change in the value of average molecular weight of polyacrylamide was shown in Table 2. The change of molecular weight is similar to the tendency of the storage and loss modulus of the hydrogel that was analyzed with the rheometer. This result is due to polymer crosslinking or scission by ionizing radiation^35^. When the radiation exposure is low, chemical crosslinking dominate, hence the modulus and molecular weight increase, and the self-healing efficiency decreases with an increase in chemical crosslinking. Then, as the radiation exposure increases, scission dominate. As a result, the modulus, molecular weight, and self-healing efficiency also decrease as shown in Fig. 3d.

**Table 2:** Molecular weights (GPC) of PAAm with different radiation exposures.

Radiation dose	*M*_n_^a^ (kDa)	*M*_w_^b^ (kDa)	PDI^c^
0 h	95.24	1204.4	10.76
0.5 h	108.6	1173.39	10.81
1 h	110.06	1008.94	9.17
1.5 h	167.01	1342.65	8.04
2 h	123.42	1319.55	10.7
2.5 h	143	1272.7	8.9
3 h	135.1	1344.83	9.96

^a^Number-average molecular weight (*M*_n_).

^b^Weight-average molecular weight (*M*_w_).

^c^Polydispersity index (*M*_w_/*M*_n_) determined by GPC with NaNO_3_ solvent and PEG/PEO standards.

## Conclusion

In this study, we have presented a soft radiation shield made using hydrogel-metal composites that are highly stretchable and self-healing. By physically crosslinking polyacrylamide with clay, the hydrogen bond between the clay and polyacrylamide allowed for a high self-healing performance of the hydrogels without external stimulation. Since the self-healing property is determined by several factors that affect diffusion, we investigated how the self-healing efficiency changed with different conditions. As a result, we could verify that a high temperature, sufficient healing time and proper hydrogel composition are major factors in the self-healing efficiency. Moreover, the radiation shielding performance was effectively confirmed via gamma ray transmittance measurements. The strong radiation shielding capacity of synthesized hydrogel composites was comparable to that of concrete, but the self-healing efficiency tended to decrease with a longer radiation exposure time. To analyze this tendency, chemically crosslinked hydrogels and a polyacrylamide were fabricated. Then, the hydrogels and polyacrylamide were exposed to gamma radiation and analyzed using a rheometer and a GPC. In this way, the similar tendency to change of molecular weight, storage and loss modulus was investigated due to chain scission or crosslinking caused by radiation. We expect this study presenting future opportunities toward radioactivity management to be useful in replacing heavy materials with soft materials to enhance efficiency and versatility.

## Method

### Gel fabrication

The polyacrylamide–Laponite–lead dioxide nanoparticle hydrogel composites were synthesized by using AAm (Sigma, A8887) as monomers for the polymer network, Laponite XLG (Clay; BYK Additives) as a physical crosslinker, potassium persulfate (KPS; Sigma, 216224) as radical initiator, *N*,*N*,*N*′,*N*′-tetramethylethylenediamine (TEMED; Sigma, T7024) as an accelerator, and PbO_2_ (Alfa Aesar, 12483) as a radiation attenuating agent. The gels were prepared by dissolving various molar concentrations of AAm monomer and clay into deionized water. The solution was mixed sufficiently to form a homogenous solution. Then, a PbO_2_ solution of 3.23 M was added to the AAm/clay solution and degassed under sonication (SONICS & MATERIALS, VCS-130). KPS 0.0037 M and TEMED 0.0056 M were added to initiate polymerization, and the hydrogel-metal composite solution was poured into an acrylic mold. The mold was sealed with a glass plate to prevent oxygen exposure that would inhibit the polymerization by reducing radicals^36^. The solution was cured at room temperature for 48 h to obtain a stabilized soft shield.

### Cut-and-heal tests for the hydrogel composite

A geometry of the sample with 50 mm in length, 15 mm in width and 2 mm in thickness was used. For self-healing test, the middle of tensile specimens was cut with a razor blade. After cutting, the separated hydrogel composites were healed by keeping the cut surfaces in contact for various periods (1–6 h) at various temperatures (25–55 °C) in an oven. Mechanical tensile tests were performed to measure the efficacy of the healing. A geometry of the sample with 50 mm in length, 15 mm in width and 2 mm in thickness was used. The specimens were mounted to roller grips and were stretched with a tensile machine (Instron, 3343) with a 50 N capacity load cell at room temperature. The initial length of the specimen between the grips was 2 mm and the extent to which the specimen was stretched from the initial length was measured. The stretch rate was 100 mm s^−1^.

### Radiation attenuation test

The hydrogel composites with various thickness (5 mm–20 mm) were loaded in a cylindrical nylon mold. The inner diameter of the nylon mold and hydrogel composites was 25 mm. The nylon mold was shielded with a cylinder of lead as shown in Fig. 3a so that the radiation could be exposed to the gel constantly. Γ-ray radiation was transmitted from a Co-60 (Nordion Inc., Canada) source, and the transmittance was measured using a γ-ray detector (NanoDot Dosimeters; LANDAUER Inc., USA). The γ-ray exposure was conducted for 10 min.

### Rheometer and gel permeation chromatography (GPC) analysis

Chemically crosslinked hydrogels were prepared to measure the shear modulus of the hydrogels. The chemical gels were prepared with MBAAm (0.062 wt. % to AAm) as a crosslinker. Other compositions were the same as for Laponite—hydrogel composites without PbO_2_, and polymerization was carried out for 2 days at room temperature. The shear modulus of the hydrogels was measured using a rheometer (TA instruments, DHR-2) with an angular frequency from 1 to 100 rad/s at 25 °C. For a gel permeation chromatography (GPC), polyacrylamide was synthesized without a crosslinker. Polymerization of polyacrylamide was carried out for 2 days at room temperature. The change in the molecular weights of the polyacrylamide was tracked by the duration of the gamma ray exposure (0.5 h–3 h). GPC instrument (HLC-8320 EcoSEC) was used with a 0.1 M NaNO_3_ solvent. The flow rate and temperature were fixed as 1.0 ml/min and 40 °C, respectively. Polyethylene (PEG) / Polyethylene oxide (PEO) standards were used to analyze the data in EcoSEC software.
